# Learning infectious disease epidemiology in a modern framework

**DOI:** 10.1371/journal.pcbi.1005642

**Published:** 2017-10-19

**Authors:** Andreas Handel

**Affiliations:** Department of Epidemiology and Biostatistics and Health Informatics Institute and Center for the Ecology of Infectious Diseases, The University of Georgia, Athens, Georgia, United States of America; Genome Quebec, CANADA

This is a *PLOS Computational Biology* Education paper.

## Summary

Modern infectious disease epidemiology makes heavy use of computational model–based approaches and a dynamical systems perspective. The importance of analyzing infectious diseases in such a way keeps increasing. However, infectious disease epidemiology is still often taught mainly from a medical and classical epidemiological study design (e.g., cohort, case-control) perspective.

While textbooks and other resources that teach a model-based approach to infectious diseases exist, almost any such teaching material requires students to work with mathematical models and write computer code. This is a significant barrier for students who do not have a strong mathematical background or prior coding experience, which applies to many students in public health and related biomedical disciplines. It limits the number of students who can or want to engage with infectious disease epidemiology by using modern, systems modeling–based approaches. New tools and approaches are needed to reach a wider audience and allow students to learn concepts such as the reproductive number, herd immunity, critical community size, and the population-level impact of interventions from a dynamical systems and model perspective, without the obstacles of coding or having to formulate and analyze differential equations.

Here, I describe a new software package for the widely used R language that allows individuals to explore and study concepts of infectious disease epidemiology by using a modern, dynamical systems model framework, without the need to read or write computer code. The package includes documentation and material to serve as a stand-alone tool—supplemented as needed with provided references—for students to get an introduction to important modern infectious disease concepts. The package is built in a modular way that allows a student to seamlessly continue on their journey of learning infectious disease modeling if they choose to do so. The different ways to use the package are described in detail, and examples are provided.

## Background and motivation

Epidemiology has its roots in the study of infectious diseases. While modern epidemiology deals with a host of different health-related topics, infectious diseases are still an important component. The classical study approach of epidemiology, based on randomized controlled trials, cohort studies, case-control studies, and related study designs, can be successfully applied to infectious diseases.

However, this framework of study generally does not account for a crucial aspect of infectious diseases, namely, the nonindependence between individuals. Accounting for interactions between hosts is required to understand important infectious disease concepts such as population-level immunity thresholds, critical community size, or indirect effects of interventions.

On the research side, the importance of such interactions has long been appreciated and is at the core of the infectious disease modeling paradigm, in which one builds and analyzes a system of interacting components (e.g., susceptible and infected hosts). Such systems approaches based on models have a long history in infectious disease epidemiology [1,2]. The importance of computational methods for the study of infectious diseases continues to increase. However, teaching such computational approaches to students in public health, medicine, and related areas remains a challenge [3].

While resources (e.g., textbooks [1,4,5]) that teach this modern approach to infectious diseases exist, one of the main obstacles is the requirement to write computer code, which tends to be a barrier for students who do not have any prior coding experience. In my own experience teaching a course entitled “Modeling Infectious Diseases,” students often struggled with the mathematical and especially the coding aspects, which interferes with their ability to understand the scientific concepts. If a student intends to become an infectious disease modeler, they undoubtedly need mastery of the mathematical and coding components. However, many students in public health and related disciplines do not intend to become modelers. They will, however, in their future career, likely become consumers of results from modeling studies. Thus, they could still greatly benefit from learning infectious disease epidemiology from the perspective of a dynamically interacting system, without the obstacles of coding and differential equations.

Good examples of ways to teach modern infectious disease epidemiology concepts without requiring students to have computational or mathematical skills are some recent online courses, most notably the course “Epidemics—the Dynamics of Infectious Diseases” [6], developed by faculty from Penn State University, and the course “Epidemics,” developed by faculty from Hong Kong University [7]. While these courses are excellent resources, they consist of passive learning, with students mainly watching lectures. Active learning, i.e., directly interacting with the material, often leads to better learning outcomes [8,9]. Computer simulations are an ideal method for facilitating such active learning. Furthermore, exposure to computer simulations can provide a familiarity with such tools that will greatly enhance the students' ability to assess and integrate results from models into their future decision-making, even if the student will never build their own model. Lastly, a gentle introduction to computational approaches can help to lower entry barriers for students on their path to becoming active modelers. To facilitate these goals, I developed the R package *Dynamical Systems Approaches to Infectious Disease Epidemiology* (DSAIDE), which allows students to learn infectious disease concepts by using a modern computational and modeling approach while not requiring—though allowing and encouraging, as described below—students to read or write computer code.

## Intended audience and goal of the package

The audience for the DSAIDE package are individuals interested in understanding infectious disease spread and control on the population level from a dynamical systems and modeling perspective. The package was originally built to complement a course on infectious disease epidemiology from a dynamical systems perspective. However, the documentation contained within DSAIDE strives to be detailed and self-contained enough to allow a motivated student to use DSAIDE and learn the topics covered by the package on their own. Any knowledge gaps can be filled by reading the provided references. For more advanced students who are comfortable with some level of coding, the package can be used as described in “Level 2” and “Level 3” below, either on its own or as a complement to a course on infectious disease modeling.

## Package description

The package currently consists of 12 simulations (hereafter referred to as “apps”) that allow for the exploration of different topics in infectious disease epidemiology. Table 1 lists all available apps (as of the time of this publication) and provides brief explanations for each. Each app is meant to be fully self-explanatory and contains a description of the model, a list of tasks the user could try, and information on further details and readings. All currently available apps are implemented as compartmental dynamical models, either deterministic using ordinary differential equations (deSolve package [10]) or stochastic using a Gillespie-type approach (adaptivetau package [11]). Using the functionality of the R shiny package [12], a graphical user interface is wrapped around each underlying simulation. This allows students to explore the models and infectious disease epidemiology concepts without the need to write any code. At the same time, the package is structured in a modular way that allows interested students to directly interact with and modify the underlying simulations in a step-wise manner, as described below.

**Table 1:** Apps currently available in DSAIDE.

App_Name	Model	Topic_Covered
ID Dynamics Intro	3-compartment (SIR) ODE model.	A first introduction to a simple compartmental SIR model. Allows simulation of a single outbreak for different parameter and initial condition settings.
Characteristics of ID	6-compartment ODE model.	The potential role of different disease states (e.g., presymptomatic, asymptomatic, symptomatic) on ID dynamics.
ID Patterns	6-compartment ODE model. Includes natural births and deaths, waning immunity, and seasonality in transmission.	Different ID patterns (single outbreak, oscillations, steady states).
Direct Transmission	3-compartment ODE model. Births, deaths, and waning immunity are included.	The differences between density-dependent and frequency-dependent transmission and their impact on ID dynamics.
Environmental Transmission	4-compartment ODE model. Includes explicit environmental stage. Natural births and deaths are included.	The impact of environmental shedding, decay, and transmission.
Vector Transmission	5-compartment ODE model. Includes susceptible and infected vectors and their dynamics. Births and deaths for vectors and waning immunity for hosts are included.	Exploration of a simple vector-borne transmission model.
Reproductive Number	3-compartment ODE model. Includes vaccination of population at the beginning of the simulation. Births, deaths, and waning immunity are included.	The reproductive number concept and how to estimate it from (simulated) data.
ID Control	9-compartment ODE model. An environmental and 2 vector stages as well as 6 host stages.	The impact of different control measures for different types of ID.
Host Heterogeneity	6-compartment ODE model. 2x SIR for 2 different hosts.	The impact of host heterogeneity and core groups on ID dynamics and control.
Stochastic Dynamics	4-compartment (SEIR) stochastic model. Births, deaths, and waning immunity are included.	Stochasticity of ID dynamics, the phenomenon of ID extinction.
Evolutionary Dynamics	5-compartment stochastic model. Untreated and treated hosts infected with wild-type, and hosts infected with a resistant strain.	Interaction between drug treatment and evolution/emergence of drug resistance.
Multi-Pathogen Dynamics	9-compartment ODE model with basic SIR dynamics for 2 pathogens and coinfection.	Infection dynamics for 2 pathogens.

Abbreviations: DSAIDE, Dynamical Systems Approaches to Infectious Disease Epidemiology; ID, infectious disease; ODE, Ordinary Differential Equation; SEIR, Susceptible-Exposed-Infected-Removed; SIR, Susceptible-Infected-Recovered.

## Installing and running the package

The package is installed like any other R package. Following are quick-start instructions:

- Install R from https://cran.r-project.org/.
- Optional, recommended: install RStudio from https://www.rstudio.com/.
- Open R/Rstudio, install the package by typing install.packages('DSAIDE') into the R console.
- Load the package with library('DSAIDE').
- Call the main menu by typing dsaidemenu() into the R console.
- You are ready to explore!

Note: The package is developed and hosted publicly on Github at https://github.com/ahgroup/DSAIDE. The Github repository can be used as an alternative to CRAN to install and test the most up-to-date version of the package (which could possibly be buggy). See the package Github site for more information and to submit bug reports, feature requests, etc.

## Using the package

The following sections describe the main ways for students to interact with the DSAIDE package. The idea is that everyone starts at level 1, and then, depending on needs and interests, they can decide whether to move on to the next level.

### Level 1: Interactive use through the graphical user interface

**Fig 1:**
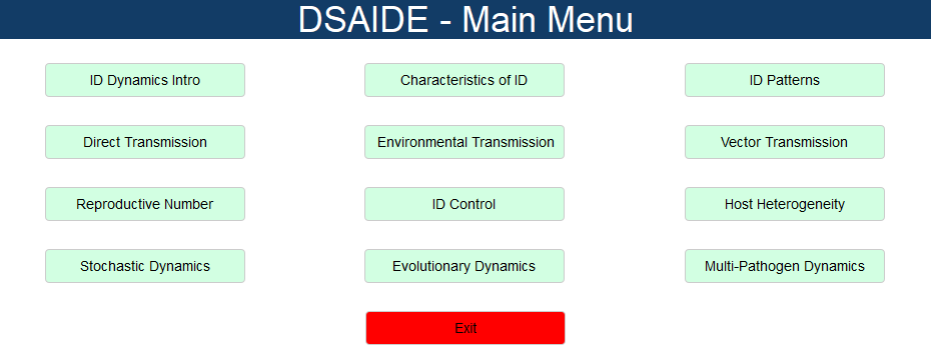
Main menu of the DSAIDE package. DSAIDE, Dynamical Systems Approaches to Infectious Disease Epidemiology.

The interactive exploration of the models and infectious disease concepts through the graphical user interface is the main intended use of this package. Once the package is loaded, the main menu is started by executing the command dsaidemenu() in the R console. This will bring up a graphical menu (Fig 1), from which the user can choose apps to explore specific infectious disease topics.

**Fig 2:**
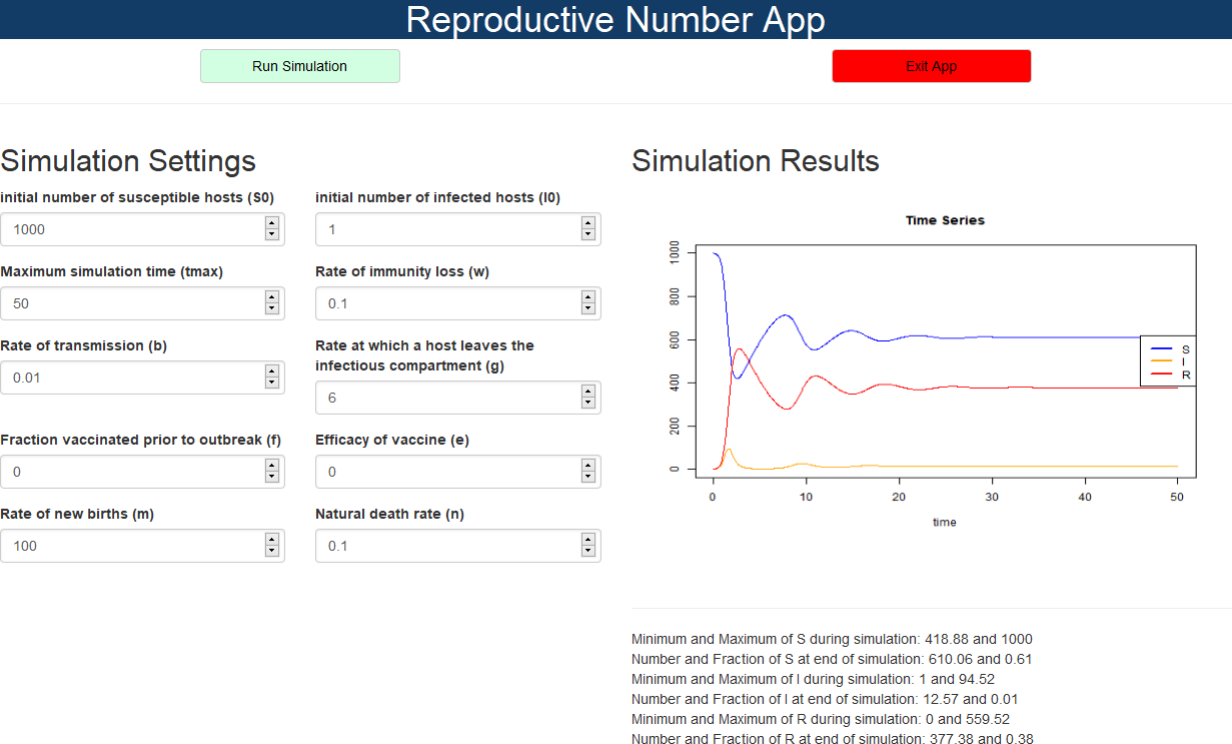
Graphical interface for the reproductive number app. Inputs are on the left; outputs in the form of graphs and useful numbers (e.g., maximum of each compartment during the simulation) are provided on the right. Below this is the documentation for the app; an example is shown in the next figure. S, Susceptible; I, Infected; R, Recovered.

The user interacts with each app through a graphical interface consisting of input boxes to set model parameters and other settings and output in the form of graphs and text. Fig 2 shows a screenshot of one of the apps.

**Fig 3:**
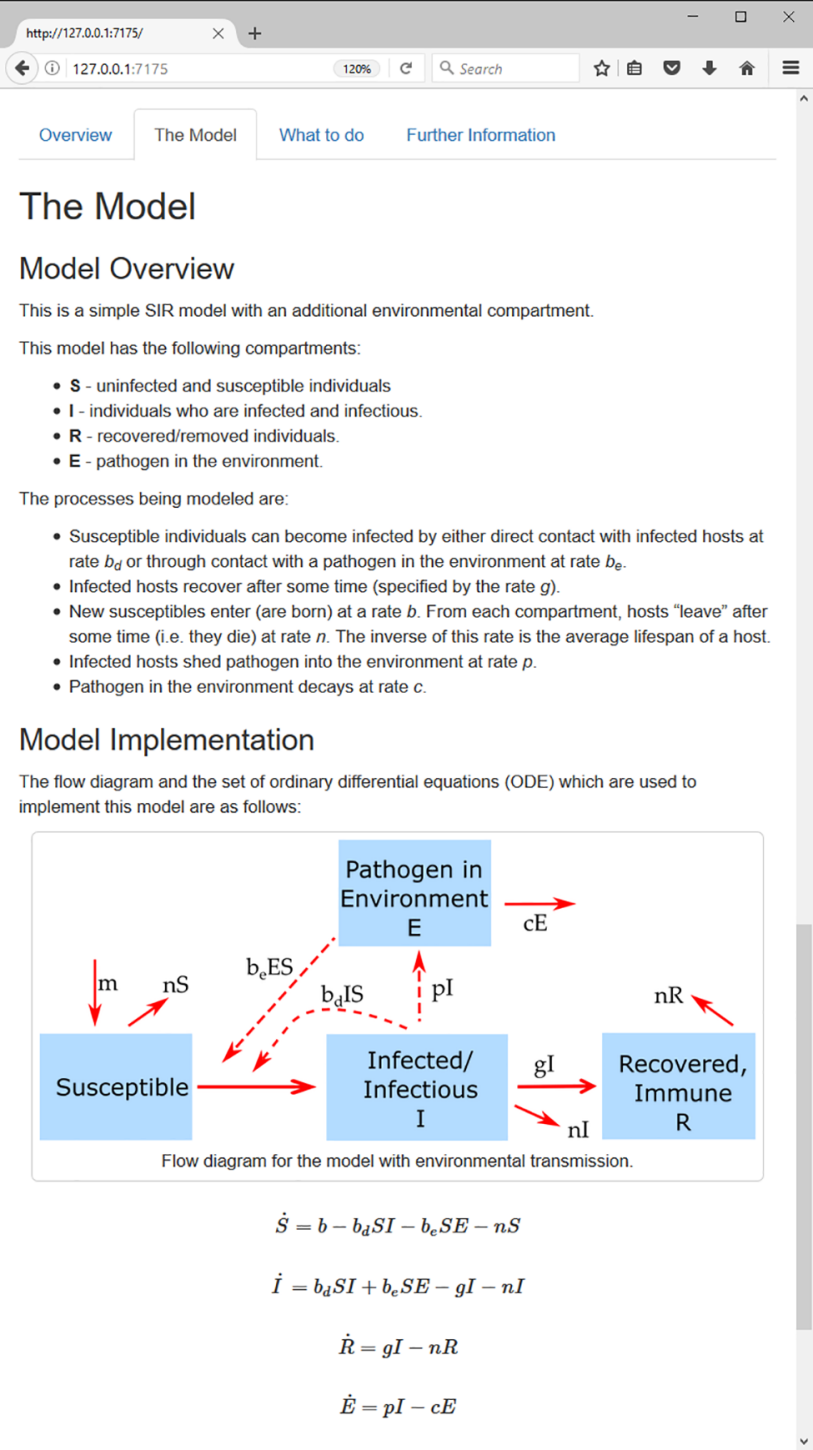
Documentation tabs for the environmental transmission app. ODE, Ordinary Differential Equation; SIR, Susceptible-Infected-Recovered.

Below the input and output areas are several tabs that contain detailed information for each app. The main tabs are the “Model” and the “What to do” tabs. The former describes the model, including showing the flow diagram and equations for the model. The latter contains a list of suggested tasks to learn about the topic covered by the app. Fig 3 shows a screenshot of some of the documentation contained in one of the apps.

After exploring an app, the user returns to the main menu and eventually exits the main menu and closes the R session. No code needs to be written. The user can fully focus on learning the infectious disease topics and concepts covered by the different apps.

### Level 2: Directly interacting with the simulation functions

The interaction with DSAIDE described in the previous section is the main intended use of the package. However, it is easy for a student to gently proceed from having no interaction with the code to writing a few lines of additional code. To facilitate direct interaction with the code, the simulation model underlying each app is a stand-alone function. This simulator function can be called directly, without going through the graphical interface. The “Further Information” tab of each app provides the name of the corresponding simulator function and a brief description of how to interact with it.

For instance, for the first app, “ID Dynamics Intro,” the underlying simulator function is called simulate_introduction.R. The user can learn about the inputs and outputs of the function by looking at its documentation (typing help("simulate_introduction") at the R console). For this function, one can specify the initial number of susceptibles and infected, the duration for which the simulation should be run, and the infection and recovery parameters. Unless explicitly specified, the models do not have inherent time units. Instead, those are set by the user based on unit choices for the model parameters. It is important to ensure that all parameters are expressed in the same time units, e.g., days or months (or the inverse of those units for the rate parameters). Each parameter has some default. The user can modify those default settings. For instance, one can call the simulator with the following settings, overwriting the defaults:

result <- **simulate_introduction**(S0 = 500, I0 = 5, tmax = 100, g = 0.1, b = 1/2500)

**Fig 4:**
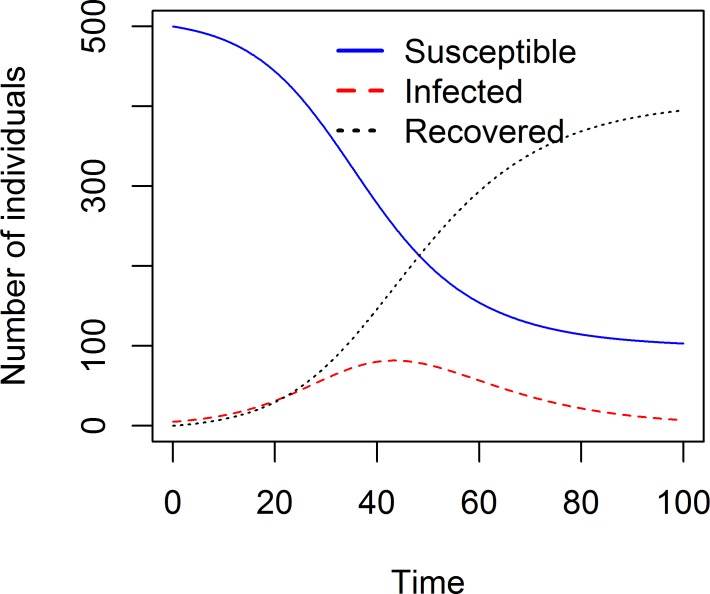
Dynamics of susceptible, infected, and recovered during a single outbreak, obtained by a direct call to the simulate_introduction() function.

Calling the simulation function runs the underlying dynamical model, here a simple 3-compartment Susceptible-Infected-Recovered (SIR) model implemented via ordinary differential equations and described in the “Model” section of the app. The simulation function produces and returns time series for the dynamics of each of the variables that are tracked. Users can produce their own plots, e.g., plotting susceptible, infected, and recovered individuals as a function of time with the code provided below. Fig 4 shows the resulting plot.

ytext = "Number of individuals";

**plot**(result[,"time"],result[,"S"],xlab = 'Time',ylab = ytext,type = 'l',col = 'blue',ylim = **c**(0,500))

**lines**(result[,"time"],result[,"I"],xlab = 'Time',type = 'l',col = 'red',lty = 2)

**lines**(result[,"time"],result[,"R"],xlab = 'Time',type = 'l',col = 'black',lty = 3)

legendtext = **c**('Susceptible','Infected','Recovered')

**legend**("top",legendtext,lty = **c**(1,2,3),lwd = 2,bty = "n",col = **c**('blue','red','black'))

Calling the simulation functions directly allows additional exploration of the models. For instance, if one wanted to systematically explore the behavior of a model for different values of some model parameter, one would need to do this manually if run through the graphical interface. By calling the function directly, this can be automated. As an example, the following lines of R code show a loop over different values of the recovery rate. At each value, the peak of the outbreak is recorded.

gvec = **seq**(0.01,0.3,by = 0.01) *#values of recovery rate*, *g*, *for which to run the simulation*

peak = **rep**(0,**length**(gvec)) *#this will record the peak values for each g*

for (n in 1:**length**(gvec))

{

  *#call thesimulator function with different values of g each time*

  result <- **simulate_introduction**(S0 = 500, I0 = 5, tmax = 200, g = gvec[n], b = 1/2500)

  peak[n] <- **max**(result[,"I"]) *#record max number of infected for each value of g*

}

**Fig 5:**
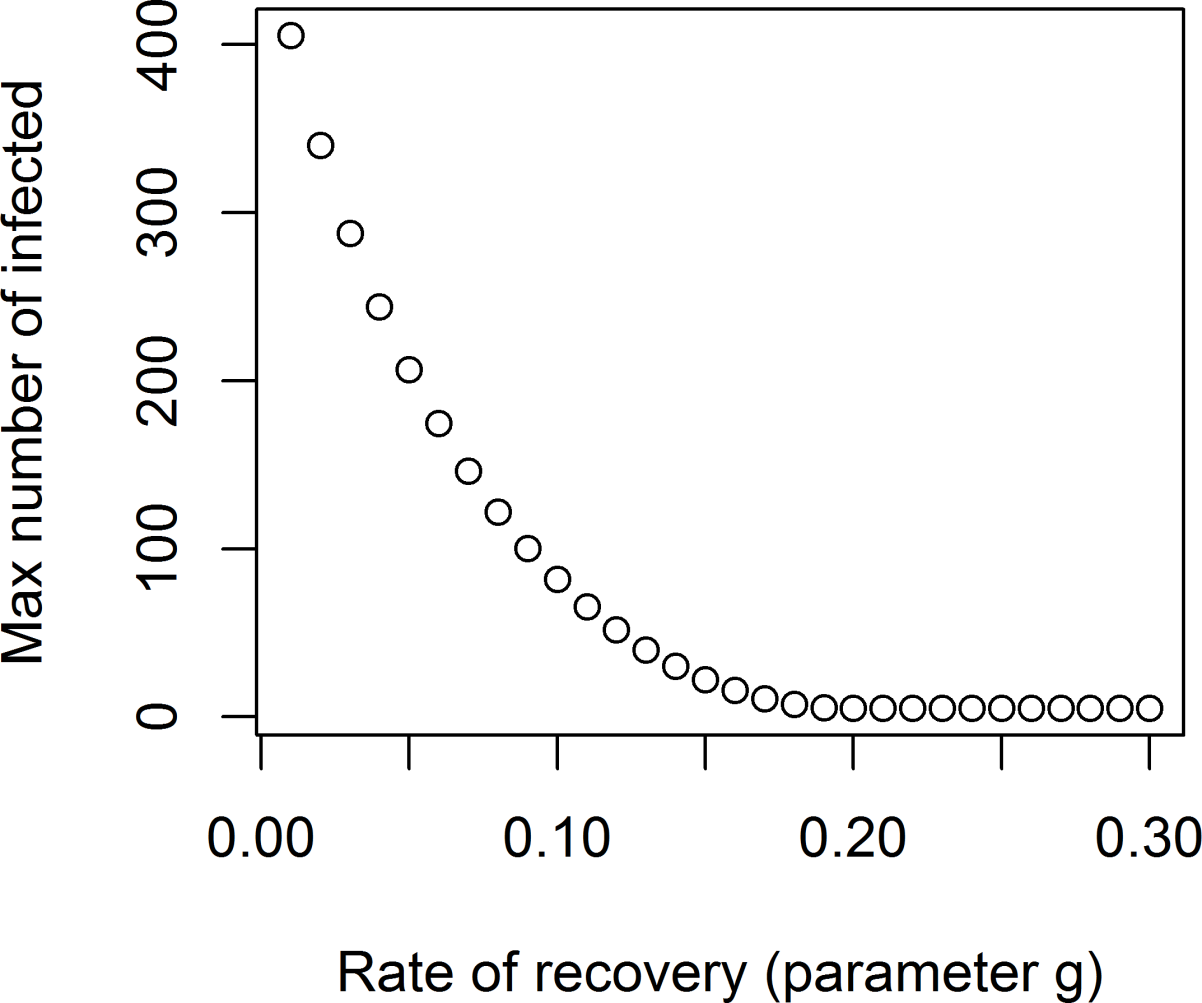
Peak of the outbreak as a function of recovery rate. This plot is obtained by wrapping the simulate_introduction() function into a loop for different values of the recovery time parameter.

Fig 5 shows the resulting plot. (The R code to plot this figure is omitted to save space).

By using this “Level 2” approach, the user can wrap their own code around the existing simulator functions and easily explore questions and scenarios that would be impossible or tedious to explore through the graphical interface. This provides a lot more flexibility. It requires writing some, but rather minimal, R code to interface with the supplied simulator functions. Once students have mastered this level and gained some coding proficiency, they can continue to the next level if interested.

### Level 3: Modifying the simulation functions

In addition to interacting with the simulation functions, the user can directly access and modify them. To make this easy, copies of all simulator functions are in a sub-directory called “simulatorfunctions” inside the DSAIDE package folder. The exact location of this folder depends on the settings for the R libraries but should be easy to locate. Each function starts with simulate_. Because these functions are copies of the ones used to run the code in the DSAIDE package, the user could edit them without breaking the package. Even so, to ensure any modifications made by the user are not overwritten if, for instance, the package is being reinstalled, I recommend copying these functions to a different folder.

Each simulator function is well documented. Some basic to intermediate level of R coding experience is required to successfully modify the functions. A simple example follows. Assume that one wants to modify the SIR model encoded in simulate_introduction.R and include waning immunity, with recovered returning to the susceptible class at rate *w*. After finding the file and making a copy (let's call the modified function mysimulator.R), modify the following lines of code of the mysimulator.R file as follows:

old:

simulate_introduction <- function(S0 = 1000, I0 = 1, tmax = 300, g = 0.5, b = 1/1000)

new:

mysimulator <- function(S0 = 1000, I0 = 1, tmax = 300, g = 0.5, b = 1/1000, w = 0)

old:

pars = **c**(b = b, g = g);

new:

pars = **c**(b = b, g = g, w = w);

old:

dS = —b * S * I; *#susceptibles*

dI = b * S * I—g * I; *#infected/infectious*

dR = g * I; *#recovered*

new:

dS = —b * S * I + w * R; *#susceptibles*

dI = b * S * I—g * I; *#infected/infectious*

dR = g * I—w * R; *#recovered*

No other parts of the simulator function code need to be modified. It is now possible to explore how different rates of waning immunity impact the outbreak peak by slightly modifying the code shown above in “Level 2,” namely, by writing a loop that goes through different values for the waning immunity parameter, *w*. The following code accomplishes this:

**source**('mysimulator.R') *#to initialize the new function—needs to be in same directory*

wvec = **seq**(0,1,by = 0.02) *#values of immunity loss rate*, *w*, *for which to run the simulation*

peak = **rep**(0,**length**(wvec)) *#this will record the peak values for each g*

for (n in 1:**length**(wvec))

{

  result <- **mysimulator**(S0 = 1000, I0 = 1, tmax = 300, g = 0.5, b = 1/1000, w = wvec[n])

  peak[n] <- **max**(result[,"I"])

}

**Fig 6:**
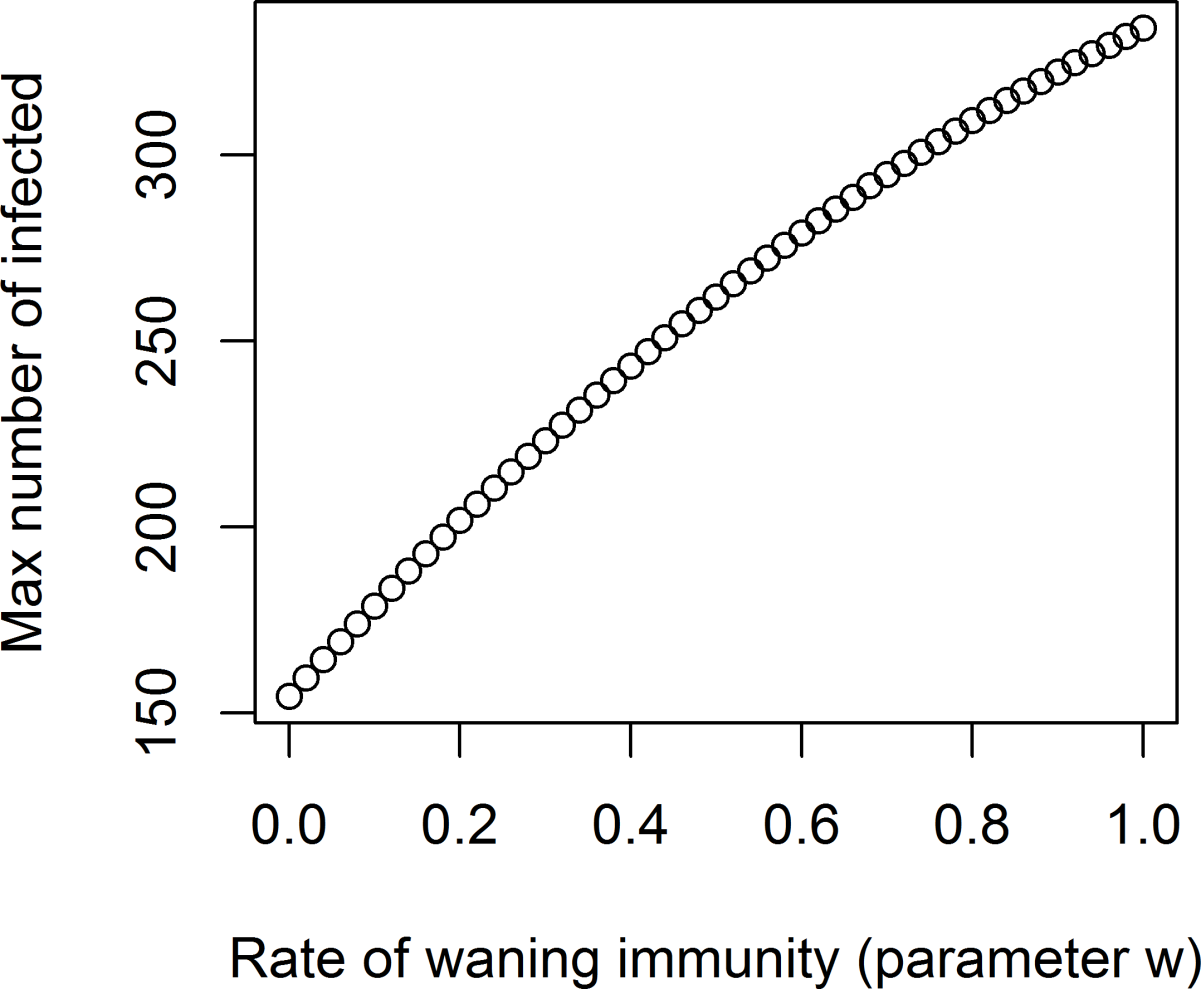
Peak of the outbreak as a function of waning immunity.

The result of this simulation is shown in Fig 6.

This approach of using the DSAIDE package allows the user to completely customize the existing code based on their own needs and interests. Once a student makes it to this level, they are on their way to becoming a modeler who builds and analyzes their own models.

### Level 4: Contributing to the package and developing new apps

It is quite likely that there are still bugs and typos in the package and its documentation; if you find some, let me know. DSAIDE is hosted on Github at https://github.com/ahgroup/DSAIDE. To submit bug reports, feature requests, and otherwise interact with the package and the package authors, please use Github if possible. DSAIDE is built in a way that (hopefully) makes it easy for others to contribute new simulations/apps. The package contains a subfolder called */docsfordevelopers* (in the locally installed version of the package, this folder is in the main package folder; on Github, it is inside the */inst* folder). The information in this folder explains the overall structure of the package and gives detailed instructions on how to create new apps. The information provided is meant to be detailed and complete enough to allow fairly easy development and contribution of new apps (or other enhancements) to the package. For any further questions, feel free to contact me via email or Github.

## Further resources

For individuals interested in tools and resources related to infectious disease epidemiology, a number of additional R packages exist. The following are packages I am currently aware of.

For learning and teaching, the EpiModel package [13] is a very good resource. This package includes network-based models, which are currently not part of DSAIDE. However, only a few simple models are accessible through a graphical user interface. While the package allows the user to simulate more detailed network models with a fairly minimal amount of coding, for a user to fully take advantage of the features provided by EpiModel, some level of coding is required. As such, the EpiModel package targets individuals who are willing to learn some coding and would like to be able to explore more advanced network-based models.

The EpiDynamics [14] R package provides somewhat similar functionality as the DSAIDE package but does not allow graphical interaction with the models and thus requires some amount of coding from the beginning.

For research purposes, the R Epidemics Consortium (RECON) [15] develops a number of R packages that are focused on providing tools to analyze infectious disease outbreaks.

A very powerful set of functions for fitting compartmental models to data is provided by the POMP R package [16]. This package requires some advanced knowledge of R and fitting. Good tutorials are provided on the package's Github site.

## Conclusion

I described the R package DSAIDE, which allows interested individuals to learn modern infectious disease epidemiology with the help of computer models but without the need to write code. The package is designed to allow easy advancement of the student toward increased flexibility in addressing questions of interest, with a concomitant (gentle) increase of required coding. Furthermore, the package allows for—hopefully rather easy—contributions of new apps. My hope is that this package will continue to grow and become a widely used and useful resource for teaching and learning modern infectious disease epidemiology.
